# The widespread presence of a family of fish virulence plasmids in *Vibrio vulnificus* stresses its relevance as a zoonotic pathogen linked to fish farms

**DOI:** 10.1080/22221751.2021.1999177

**Published:** 2021-11-18

**Authors:** Héctor Carmona-Salido, Belén Fouz, Eva Sanjuán, Miguel Carda, Christian M. J. Delannoy, Neris García-González, Fernando González-Candelas, Carmen Amaro

**Affiliations:** a Departamento de Microbiología y Ecología, & Estructura de Recerca Interdisciplinar en Biotecnologia i Biomedicina (ERI BIOTECMED), Universitat de València. Burjassot, Valencia, Spain; b Skretting Group, Stavanger, Norway; c Joint Research Unit Infection and Public Health FISABIO-University of Valencia, Institute for Integrative Systems Biology I2SysBio (UV-CSIC), Valencia, Spain; d CIBER in Epidemiology and Public Health, Madrid, Spain

**Keywords:** *V. vulnificus*, phylogeny, virulence plasmid, zoonosis, vibriosis

## Abstract

*Vibrio vulnificus* is a pathogen of public health concern that causes either primary septicemia after ingestion of raw shellfish or secondary septicemia after wound exposure to seawater. In consequence, shellfish and seawater are considered its main reservoirs. However, there is one aspect of its biology that is systematically overlooked: its association with fish in its natural environment. This association led in 1975 to the emergence of a zoonotic clade within phylogenetic lineage 2 following successive outbreaks of vibriosis in farmed eels. Although this clade is now worldwide distributed, no new zoonotic clades were subsequently reported. In this work, we have performed phylogenetic, genomic and functional studies to show that other zoonotic clades are in fact present in 4 of the 5 lineages of the species. Further, we associate these clades, most of them previously but incompletely described, with the acquisition of a family of fish virulence plasmids containing genes essential for resistance to the immune system of certain teleosts of interest in aquaculture. Consequently, our results provide several pieces of evidence about the importance of this species as a zoonotic agent linked to fish farms, as well as on the relevance of these artificial environments acting as drivers that accelerate the evolution of the species.

## Introduction

*Vibrio vulnificus* is a multi-host pathogen that inhabits marine and estuarine ecosystems in tropical, subtropical and temperate zones [1]*.* Currently, its geographic distribution is expanding to traditionally colder areas due to global warming [2]. The pathogen causes a series of diseases with multiple clinical manifestations, known as vibriosis [3,4]. Although human vibriosis has been more thoroughly studied than that of fish, studies in eels suggest that the pathogen infects animals by adhering to the gill or intestinal mucus and provoking a local inflammatory response that allows it to invade the blood and cause death by haemorrhagic septicaemia [4]. Remarkably, both human and fish vibriosis can lead to sepsis and death depending on several risk factors which, in fish, are related to water temperature and salinity [4] whereas in humans to elevated blood iron levels [5]*.* In addition, some cases of secondary septicemia transmitted from diseased fish to humans have been reported, making *V. vulnificus* the only vibrio recognized as a true zoonotic agent [6,7]*.* However, because zoonotic cases are so rare, this pathogen is mostly known as a foodborne pathogen or as a marine flesh-eating bacterium.

Classically, the species has been subdivided into three biotypes, all including environmental and clinical strains, that differ in some biochemical and serological features [8–10]*.* However, this subspecific classification system does not reflect the true variability of the species, as many strains cannot be classified into any of these biotypes. Recently, Roig et al. [11] proposed a new subspecific classification system based on the genetic variability (single nucleotide polymorphisms, SNPs) of the core genome. According to this new classification, *V. vulnificus* is divided into five lineages (denoted L1 to L5) plus a pathovar (pv. *piscis*) within L2 that groups virulent strains for fish. The distinctive feature of pv. *piscis* strains is that they possess a fish virulence plasmid (pFv) that contains two genes that, when deleted independently, they turn the bacterium practically avirulent for fish while retaining its virulence for mice [12,13]. These genes encode a “survival in fish blood kit” formed by two iron-regulated outer membrane proteins, Fpcrp (fish phagocytosis and complement resistance protein, formerly *vep07*) [13] and Ftbp (fish transferrin-binding protein, formerly *vep20*) [12]. In contrast, virulence factors that damage host tissues are all chromosomal and appear to be involved in both human and fish vibriosis [3,4,14,15]. Interestingly, pFv can be transmitted by parasitizing a conjugative plasmid (pConj) widely distributed in the species [16].

Roig et al. [11] concluded that the pFv had probably been acquired several times in fish farms by different clones that were amplified after successive outbreaks giving rise to the clades currently isolated from diseased fish: L2-clade A, L2-clade E (or serovar E [Ser E]), and L2-clade I. Remarkably, L2-clade E was the first to be isolated [8] and it groups all zoonotic strains described to date [11]. Consequently, the authors hypothesized that fish farms may play an important role in the evolution of the species by facilitating the emergence of new potentially zoonotic groups, as occurred with L2-clade E.

To test this hypothesis, we used recent isolates from vibriosis outbreaks together with control strains belonging to clades and lineages previously described in a series of genotypic, phenotypic and functional assays, as well as in phylogenetic and genomic studies. Our results suggest that previously studied lineages (L3, formerly biotype 3 [9] and L5, formerly Clade B [17], both clonal), and clades (L1-clade A [18]) together with a new clade (L1-clade T), described in this work, belong to pv. *piscis*, as all were virulent to fish and harboured a pFv-related plasmid containing the gene markers *ftbp* and *fpcrp*. None of the above groups had been linked to vibriosis in fish or to zoonosis cases [9,17,18], but all include human clinical isolates, demonstrating their zoonotic nature.

## Material and methods

Schemes of the general procedure as well as additional information on the methodology are shown in Supplementary Figures 1 and 2 as well as in Supplementary File 1.

**Figure 1.**
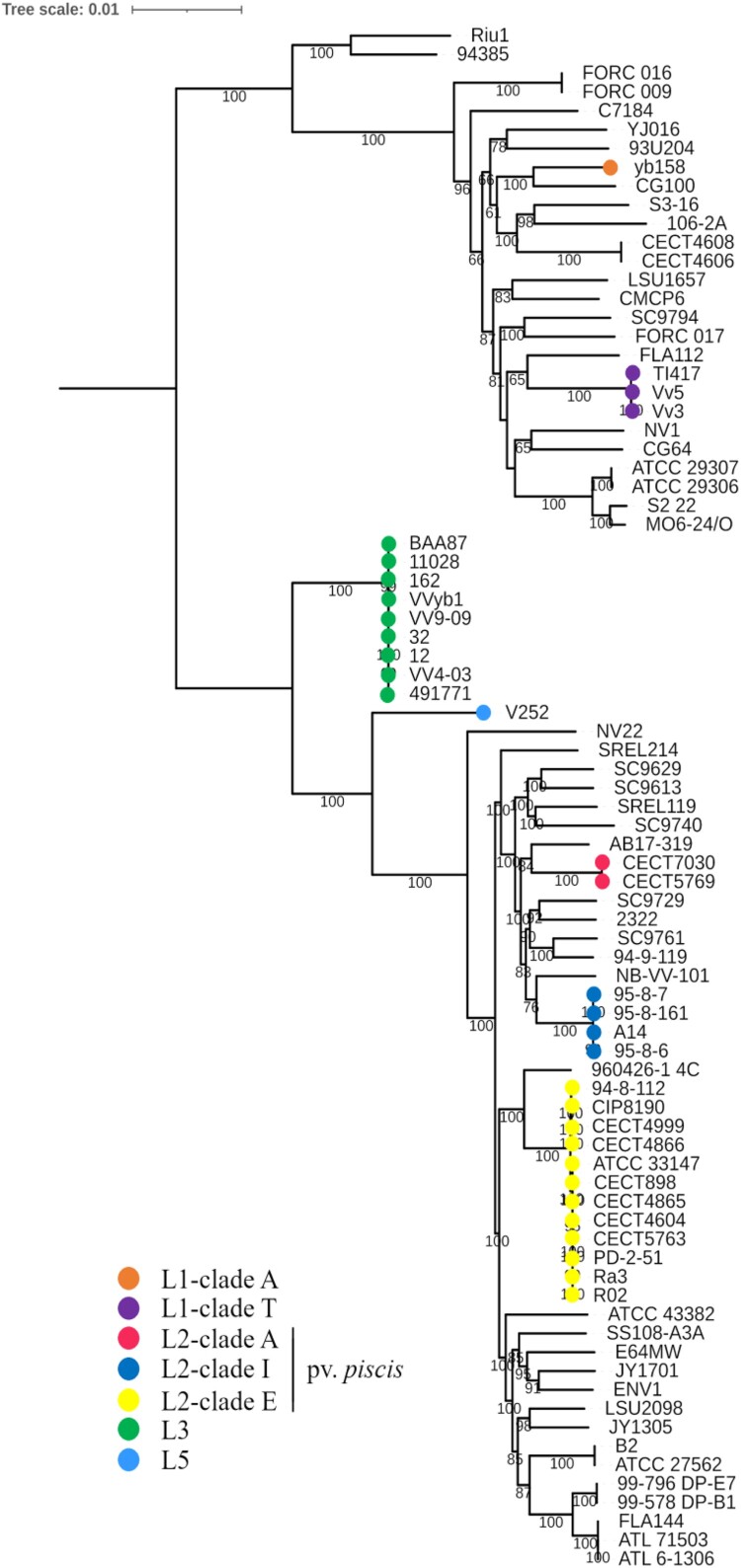
*V. vulnificus* phylogeny. The phylogenetic tree was reconstructed using the maximum-likelihood method and the generalized time-reversible model (GTR + F+R5) of evolution. Bootstrap support values from 1000 replicates are indicated in the corresponding nodes as percentages. L, lineage.

**Figure 2.**
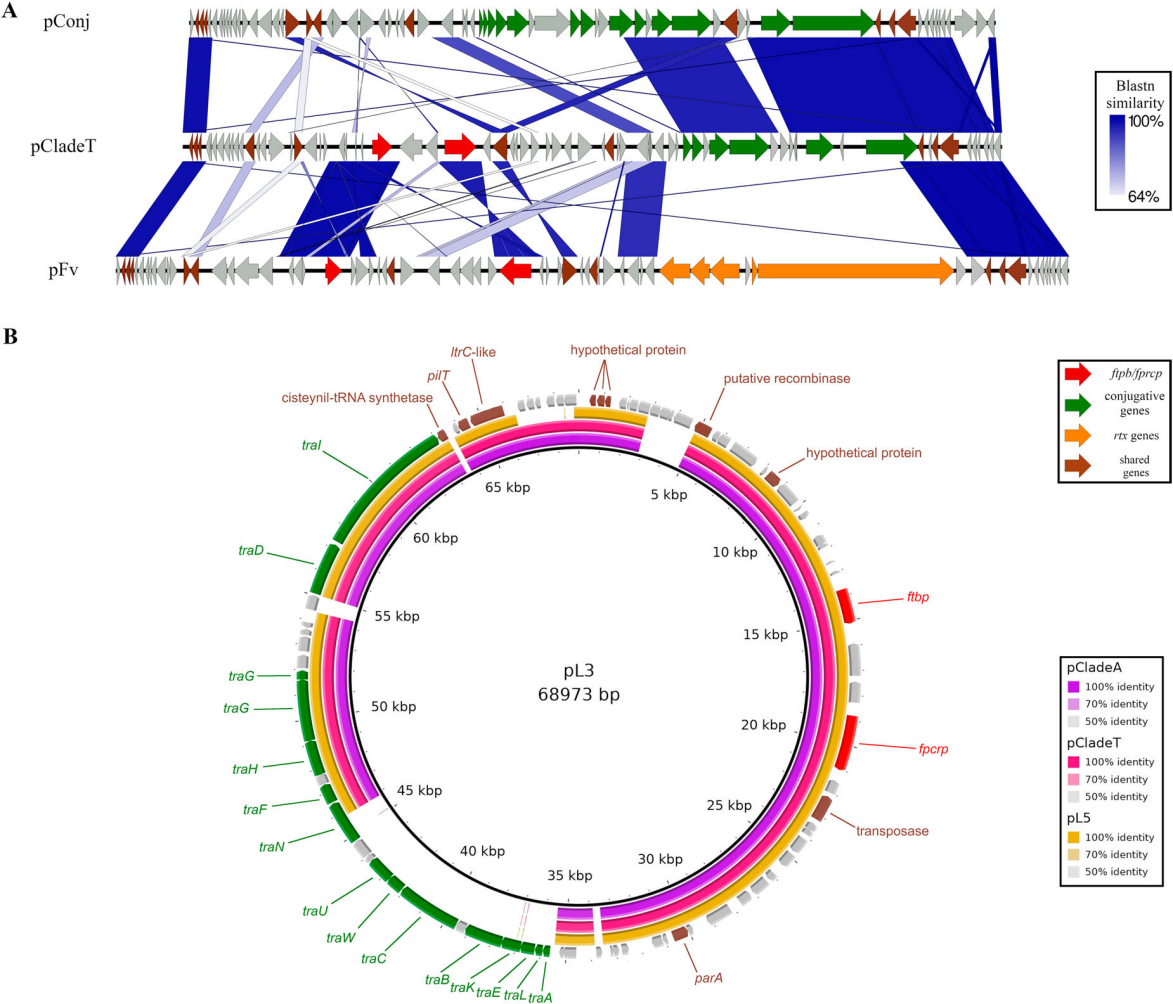
Plasmids in pv. *piscis* lineages and clades. The genes for conjugative transference, the survival in fish blood kit (*ftpb* and *fpcrp*), and the MARTX toxin are represented in green, red, and orange respectively, while the additional genes common to all pv. *piscis* plasmids are represented in brown. (A) Linear comparison among pCladeT and the original pv. *piscis* plasmids, pFv and pConj, performed with Easyfig [38]. (B) Ring representation of the pv. *piscis* plasmids from the clades and lineages emerged in the Eastern Mediterranean. From inside to outside, pL3 (used as reference; black ring), pClade A, pCladeT y pL5. The gene annotation of the pL3 is represented in the multicoloured external ring.

***Bacterial identification and serology.*** A number of isolates from diseased tilapia showing clinical signs compatible with vibriosis arrived in the laboratory [19]. These tilapia came from several fish farms located in the Eastern Mediterranean that experienced recurrent outbreaks of vibriosis between 2016 and 2019 [19]. Pure cultures were obtained from internal organs of moribund tilapia and were identified at the species level (API-20E system [BioMerieux, Madrid, Spain] plus PCR targeting *vvhA* [20]), pathovar (PCR targeting *fpcrp* [20]), and zoonotic clade (PCR targeting *seq61* [21]) levels. Identified strains were then subtyped for evaluating their putative public health hazard (PCR targeting a polymorphism in *pilF* [PHH-PCR]) [22].

The serological group of the new isolates was determined by slide agglutination and ELISA by using bacterial O-antigens and rabbit antisera against formalin-killed cells [23]. Serum titres were calculated as the reciprocal of the highest antibody dilution giving a positive result.

***In vivo and ex vivo virulence assays.*** Virulence for tilapia and mice was performed to determine the zoonotic potential of the new isolates. In the case of tilapia, juvenile healthy Nile tilapia (mean weight 8-10 g) were infected by intraperitoneal (i.p.) injection and by immersion as previously described [24]. In case of mice, females of 6- to 8-week old (BALB/c, Charles River, France) were infected by i.p. injection as previously described [10]. The virulence score of each isolate was calculated as the lethal dose causing 50% of mortality (LD_50_) following the procedure of Reed and Muench [25]. All the assays were performed in duplicate and control groups of animals were challenged with sterile PBS.

The ability of the new isolates to cause septicemia was tested using tilapia plasma and iron-overloaded human serum**.** Fresh tilapia plasma was obtained and tested as previously described [26]. Human serum (Sigma) was supplemented with FeCl_3_ 10 μm and was distributed in microtitre plates that were inoculated with stationary-phase bacteria [13]. The assays were performed in triplicate and samples were taken at 0, 4 h (fish) and 6 h (human) post-incubation at 28°C (fish plasma assay) or 37°C (human serum assay). Viable counts were determined by drop plating on TSA-1. Bactericidal and bacteriostatic activities in serum/plasma were measured as the percentage survival of the strains.

***Genomic and phylogenomic analysis.*** Genomic DNA was extracted using GenElute^TM^ Bacterial Genomic DNA kit (Sigma). DNA integrity was checked by electrophoresis and quantified with Qubit and then, DNA was sequenced. Vv5 was sequenced with Illumina MiSeq and Oxford Nanopore MinION while Vv3 and TI417 strains were sequenced only with Illumina MiSeq. Data availability, genome description and further details are described in [19]. Briefly, library construction and sequencing of Vv5 with Illumina MiSeq were performed by SCSIE (Servei Central de Suport a la Investigació Experimental) of the University of Valencia using Illumina® TruSeq® DNA PCR-Free Sample Prep Kit following manufacturer's instructions, obtaining 250 bp paired-ends reads. In addition, library construction and sequencing of Vv5 were performed at FISABIO Molecular Epidemiology and Sequencing Service laboratories, with the Oxford Nanopore PCR Barcoding Kit (SQK-PBK004) following manufacturer’s instructions. On the other hand, library construction and sequencing of Vv3 and TI417 were performed at FISABIO Molecular Epidemiology and Sequencing Service laboratories, with the Illumina® NextSeq platform using Nextera® XT Library Preparation Kit and manufacturer’s protocols (Illumina, San Diego, USA), which generates 150 bp paired-ends reads. Quality of Illumina reads was checked using FastQC (https://www.bioinformatics.babraham.ac.uk/projects/fastqc/) and MultiQC [27]. Then, reads were filtered using Prinseq [28] with a mean quality threshold of 20 (–min_qual_mean 20) and checked again with FastQC. Long reads were evaluated and filtered with NanoPack [29]. First, read quality and length were assessed with NanoStat and NanoPlot; then, reads were filtered with a minimum length threshold of 500 nucleotides. For short reads, a *de novo* assembly was performed using SPAdes genome assembler v3.13 [30] with “careful mode” for Vv3 and TI417. For hybrid assembly of short (Illumina) and long reads (Nanopore), we used Unicycler [31] v0.4.9b with default parameters and normal mode. Assembly statistics of resulting assemblies were retrieved using Quast v5.0.2 [32]. In order to obtain the strict core of the species and plasmids, all the genomes were annotated with Prokka [33] and we used Proteinortho5 [34] to determine the subset of shared orthologous genes. Individual genes were extracted from each genome assembly with the grab_proteins.pl script. For each software, default parameters were used except where indicated otherwise.

MARTX sequences were retrieved from NCBI. Domains were annotated as previously described [35] and were used as a local database to perform a BLAST analysis. The presence of CRISPR was analysed using the CRISPRcasFinder online tool [36] (https://crisprcas.i2bc.paris-saclay.fr/). Spacer sequences were analyzed using CRISPRTarget databases [37]. Comparison of genomic regions was performed using BLASTn analysis and the results were plotted with Easyfig [38]. The genomic region surrounding the CRISPR-CAS system was checked for the presence of markers of mobile genome elements such as integrases, transposases, or phages, searching in INTEGRALL, PHASTER or comparison of %GC [39,40].

A phylogenomic analysis of the core from 80 genomes [11], downloaded from the NCBI (https://www.ncbi.nlm.nih.gov/), and the genomes sequenced in this study was performed. Sequences of genes included in the strict core were aligned with Mafft [41] and concatenated using AMAS [42]. The phylogenetic tree was reconstructed using the maximum-likelihood (ML) method with IQ-TREE [43] and ModelFinder [44] option to assess the best model that fitted our data. The model used based on the BIC criterion was GTR + F+R5; additionally, we assessed branch support with 1000 bootstrap replicates [45]. The resulting tree was visualized using the online tool iToL [46].

Phylogenetic trees for *ftbp* and *fpcrp* were reconstructed using the ML method with the Tamura 3-parameter model [47]. Support for the groupings derived in these reconstructions was evaluated through bootstrapping using 1000 replicates. In both cases, the best evolutionary model for the nucleotide sequences was selected by using MEGA X [48]. The sequences of both genes were obtained from our genome database [11] and from GenBank for *V. harveyi* (HM752246.1).

## Results

### Characterization of a new emerging group within V. vulnificus pv. piscis

All tilapia isolates were identified as *V. vulnificus* by PCR and gave the same phenotypic profile in the API20E system. Therefore, we selected four of them for further studies. Table 1 presents the results for the four isolates and control strains in every performed test.

**Table 1.** Isolation data, lineage and identification of the new isolates in comparison with those of the control strains.

	Isolation data			Identification^d^		^ ^
Strain^a^	Source^b^	Geographic location/year of isolation	Phylogenetic Lineage^c^	Species PCR/Api20E	Pathovar/clade E	Public Health Hazard
**New isolates**						
VV3, VV4, VV5	Diseased tilapia	Eastern Mediterranean /2016	?	+/*+* (99.3%)	**-/-**	**+**
TI417	Diseased tilapia	Eastern Mediterranean /2019	?	+/*+* (99.3%)	**-/-**	**+**
**Control strains**						
YJ016	Human blood	Asia/1993	L1	*+/+* (99.3%)	-/-	**+**
CECT 529^T^	Human blood	USA/1980	L2	*+/+* (99.3%)	-/-	**+**
CECT 4999	Diseased eel	Europe/1999	L2/clade E	+/¿? (54.4%)	+/+	**+**
CECT 5198	Diseased eel	Europe/2000	L2/clade A	*+/+* (99.3%)	+/-	**-**
95-8-161	Diseased eel	Europe/1995	L2/clade I	*+/+* (99.3%)	+/-	**+**

^a^ CECT, Spanish Type Culture Collection; T, type strain.

^b^ All the new isolates were recovered as pure cultures from internal organs of moribund tilapia. VV3 was isolated from farm A, VV4 and VV5 from farm B and TI417 from farm C.

^c^ Phylogenetic lineage determined by Roig et al [11]. L1 includes biotype 1 strains, L2 biotypes 1 and 2 strains and L3 biotype 3 strains. L2-Clade E includes all the zoonotic strains reported to date.

^d^ Identification of the isolates was performed at species, pathovar (*piscis*) and zoonotic clade (clade E) level by PCR targeting *vvhA* [20]*, fpcrp* (formerly *vep07*) [21] and *seq 61* [21], respectively. Positive (+), negative (-), and doubtful (¿?) identification. The value in parentheses indicates the probability of a good identification according to the API20E profile (5146105, probability 99.3%; 5006005, probability 54.4%).

^e^ The public health hazard of the strain was determined by PCR targeting a polymorphism of *pilF* [22]. Discrimination is based on the amplification of a variable region located within the gene *pilF* resulting in a 338 bp fragment.

*PCR-subtyping and serology.* The selected tilapia strains were negative for pv. *piscis*-PCR [21], positive for the PHH-PCR *pilF* [22] (Table 1) and serologically identical and distinguishable from previously described serovars (Supplementary Table 1). Therefore, they represent a new serovar within the species that did not belong to pv. *piscis* for which the name Ser T (from tilapia) is proposed.

*Virulence assays: ex vivo and in vivo assays*. To find out if Ser T was the etiological agent responsible for the outbreaks in tilapia we performed a series of *ex vivo* and *in vivo* assays with fish. The Ser T strains multiplied in fresh tilapia plasma in less than 2 h and were virulent to tilapia by both i.p. injection and immersion (Table 2), reproducing the clinical signs of natural disease (Supplementary Figure 3). These results clearly demonstrated that Ser T was the responsible etiological agent that caused the outbreaks of vibriosis in tilapia and, therefore, that they should belong to pv. *piscis*. Interestingly, Ser T strains were avirulent to eels (LD_50_>10^8^ cfu/ml by immersion challenge, data not shown) while the control strain from the zoonotic clade (L2-Clade E), originally isolated from diseased eel, was virulent to eel (LD_50_>1 × 10^6^ cfu/ml by immersion challenge [24]) but not to tilapia (Table 2), suggesting host-specificity for both groups of isolates. As expected, control strains of human origin neither grew in tilapia plasma nor were virulent to tilapia (Table 2). Furthermore, to predict the zoonotic potential of Ser T, we performed experiments of resistance to iron-overloaded human serum and virulence in mice (Table 2). All Ser T strains multiplied in iron-overloaded human serum and were virulent to mice giving values of survival percentage and lethal dosis 50% (LD_50_) similar to those of the zoonotic-clade and human-derived strains used as controls (Table 2). All these data strongly supported the hypothesis about the zoonotic potential of the new serovar. Due to the contradictory results obtained in the pv. *piscis*-PCR, we decided to sequence the genome of three Ser T representative strains.

**Table 2.** Results obtained in the in vivo and ex vivo virulence assays performed with the new isolates in comparison with those of the control strains**.**

	Resistance to ^a^		Virulence for^b^		
			Tilapia		Mouse
Strain	TP	HS + Fe	i.*p*. injection	Immersion	i.*p*. injection
Tilapia isolates					
VV3	+ (9.1 × 10^3^%)	+ (7.0 × 10^2^%)	+ (3.3 × 10^6^)	+ (3.3 × 10^5^)	+ (1.5 × 10^6^)
VV4	+ (8.3 × 10^3^%)	+ (1.1 × 10^3^%)	+ (8.4 × 10^5^)	+ (8.4 × 10^5^)	+ (9.2 x 10^5^)
VV5	+ (8.5 × 10^2^%)	+ (3.1 × 10^3^%)	+ (2.5 × 10^6^)	+ (4.2 × 10^5^)	+ (2.0 × 10^6^)
TI417	+ (9.5 × 10^2^%)	+ (1.7 × 10^3^%)	NT	+ (1.0 × 10^6^)	NT
Control strains					
CECT 4999	+ (8.5 × 10^2^%)	+ (1.9 × 10^3^%)	- (> 10^7^)	- (> 10^8^)	+ (1.0 × 10^6^)
YJ016	-(0%)	+ (4.9 × 10^3^%)	- (> 10^7^)	- (> 10^8^)	+ (1.0 × 10^6^)
CECT 529^T^	-(0%)	+ (5 × 10^2^%)	- (> 10^7^)	- (> 10^8^)	- (> 10^7^)

^a^ Resistance to tilapia plasma (TP) and iron-overloaded human serum (HS+ 10 μM of FeCl_3_ [13]) after 4 h at 28°C (tilapia) or 6 h at 37°C (human) is coded as follow: +, survival ≥100%; -, survival <100%. Data in parentheses correspond to the medium percent survival from three independent experiments.

^b^ The medium value of 50% lethal dose (LD_50_) from two different experiments is presented in parentheses as cfu/g (i.*p*. injection) or /ml (inmersion). Results are coded as follows: fish and mouse injection; - (≥10^7^), + (< 10^7^); fish immersion; - (≥10^8^) + (< 10^8^) [10, 24].

NT, not tested.

#### Genomic and phylogenomic analysis

First, we performed a phylogenomic analysis from the genes shared by our genomes and the genomes used in the analysis of Roig *et al.* [11]. The number of shared genes (strict core) was 2619, with a total of 203,554 SNPs. a number higher to that found by Roig et al. [11]. However, the corresponding phylogenetic tree presented the species divided again into 5 lineages with a topology very similar to that found by Roig et al. for each chromosome [11] (Figure 1). Ser T strains clustered (clade T), which was compatible with an ANI value close to 100% (Supplementary Table 2), within L1 (Figure 1), the lineage that grouped most strains from cases of primary sepsis in humans [11] while all pv. *piscis* clustered within L2. This result, again, suggested that Ser T strains did not belong to pv. *piscis*.

To further verify this, we searched for the pathovar marker gene (*fpcrp*) in the three sequenced genomes. We found an almost identical gene in the three genomes that differed from that previously described [21] by only 19 nucleotides out of a total of 1398, 3 of which just were located at the 3’ end of one of the primer pairs used in the PCR to identify the pathovar (Supplementary Figure 4). Consequently, we concluded that clade T effectively belonged to pv. *piscis* and designed a new PCR to identify pv. *piscis* strains. The new primer pairs are Ftbp F: 5’-AGTTTGCGGAAAAAGCACAG-3’/Ftbp R: 5’-CATTGATCGTCGTCTGAACC-3’ and amplify a fragment 392 pb.

Since *fpcrp* is a plasmid gene, we looked for the presence of plasmids in our genomes. To facilitate this task, we sequenced the genome of one of the Ser T isolates using MinION, which allowed us to obtain all the plasmid genes in a single contig (pCladeT). The plasmid was compared to plasmids pFv and pConj, previously described in pv. *piscis* [49] (Figure 2A). pFv is about 68 Kb in size and contains a complete gene cluster for the RtxA1 toxin, its post transcriptional modification, and transport (Supplementary file 2) (Figure 2A). This cluster is duplicated in chromosome II of L2-clade E strains [49]. pFv also has two genes for a toxin-antitoxin system, the fish virulence markers *ftbp* and *fpcrp* (the marker gene for pathovar). Finally, pFv encodes a series transposases and hypothetical proteins or proteins with very low similarity to known proteins (Supplementary file 2) (Figure 2A). In contrast, pCladeT has a size of 56 Kb and, apparently, is a hybrid between pConj and pFv because it contains *tra* genes with high similarity to pConj genes and genes with high similarity to those found in pFv, such as genes for the toxin-antitoxin system, genes for transposases, genes for hypothetical proteins, and, most importantly, the two genes for the “survival in fish blood kit” (Supplementary file 2) (Figure 2A). In consequence, our results suggest that pCladeT is a fish virulence plasmid. To confirm that this plasmid was present in all strains from tilapia, we performed a PCR for *ftbp and fpcrp* and found all the strains positive (Supplementary Figure 5).

**Figure 3.**
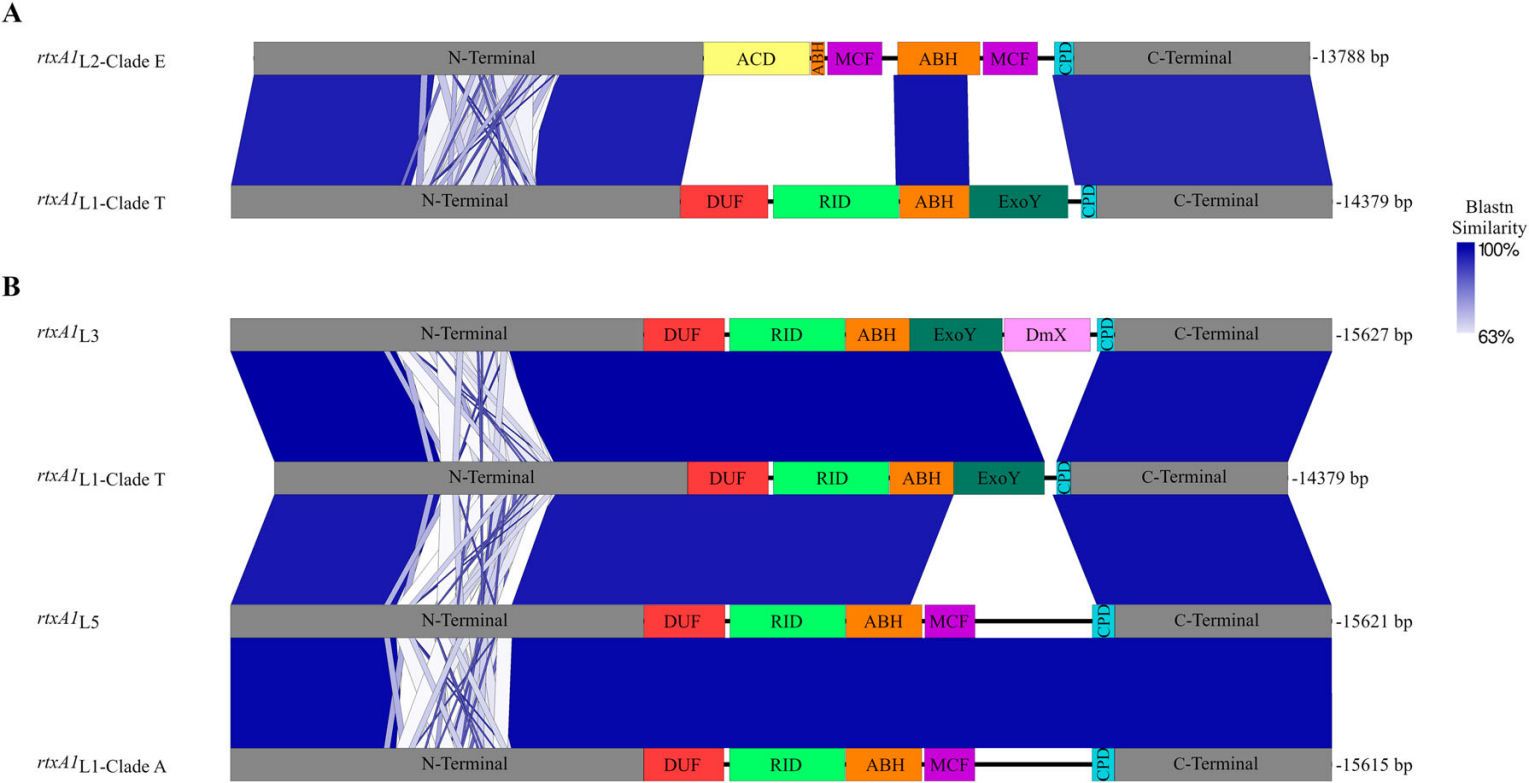
*rtxA1* gene structure. (A) Schematic representation of *rtxA1* gene from pv. *piscis* L2-clade E and L1-clade T (*rtxA1*_L2-clade E_ and *rtxA1*_L1-clade T_) (B) Comparison among the *rtxA1* genes of the clades and lineages emerged in the Eastern Mediterranean. The images were constructed with Easyfig and the blue scale indicates nucleotide Blast similarity. Carboxi and amino terminal modules are coloured in grey, cysteine protease domain (CPD) in turquoise, Domain X (DmX) in pink, ExoY-like adenylate cyclase domain (ExoY) in black, makes caterpillars floppy-like domain (MCF) in orange, alpha/beta hydrolase domain (ABH) in purple, Rho GTPase-inactivation domain (RID) in green and domain of unknown function at the first position (DUF1) in yellow.

pCladeT lacked the genes for the *rtxA1* cluster. We found the cluster in chromosome II. RtxA1 is an essential virulence factor for fish and humans in this species [14,15]. This toxin belongs to the MARTX (Multifunctional, Autoprocessive, Repeat in Toxin) family and is the main toxin of the species [14,50,51]. These are modular toxins of a very high molecular weight, with an external module that contains the amino acid repeats and an internal module that contains between 3 and 5 functional domains with different cytopathic functions. MARTX_L1-clade T_ was different to the toxin of this family described in the rest of pv. *piscis* strains (Figure 3A). The most similar toxin was that of L3, as both toxins were practically identical but that of L1-clade T lacking Dmx domain (Figure 3B).

Interestingly, a CRISPR-Cas system was identified in the L1-clade T genomes but not in the rest of *V. vulnificus* genomes, regardless of the lineage and clade. This system was practically identical to other *Vibrio* CRISPR-Cas systems, such as those corresponding to *V. cholerae* RFB05 or *V. anguillarum* PF7 (Supplementary Figure 6) but showed as a distinctive feature its location in a 45 Kb island encoding a potassium dependent ATPase, a type I restriction/modification DNA system, and a series of unknown proteins within a histidine biosynthesis operon (Supplementary Figure 6). The CRISPR-Cas_L1-clade T_ system was identified as of type I-C by the presence of a canonical *cas* operon (*cas2cas4cas3cas1cas5cas7cas8c*), a leader sequence and a characteristic canonical type I-C repeat [52]. The array contained 65 spacers, 33 of which matched some vibriophages (such as VP882 or fs2) and plasmids from different *Vibrio* species (*V. parahaemolyticus* pVPGX2, *V. vulnificus* p4810 or *V. harveyi* pLA16-1 among others) [52,53]. No marker of mobile genetic elements in the genomic region surrounding the CRISPR-Cas _L1-clade T_ system was identified. Since the protospacer targets several phages and plasmids, some of which were vibriophages and *Vibrio* plasmids, we suggest that this system protects the bacteria against attacks by phages and the entry of exogenous DNA.

### Retesting L3, L5 and L1-clade A

The L1-clade T genomes were more similar to L3-, L5- and L1-clade A genome than to the previously described pv. *piscis* genomes (Supplementary Table 2). All these groups had in common that they (i) were highly clonal, (ii) had arisen in tilapia farms in the Eastern Mediterranean, and (iii) included human clinical and environmental strains with no reported relationship to cases of zoonosis or fish vibriosis [9,17,18]. Consequently, we suspected that these groups might also belong to pv. *piscis*. To confirm this hypothesis, we characterized representative isolates from these groups by performing the same analyses that we had carried out with the L1-clade T strains.

*PCR-Subtyping and serology*. The results obtained are shown in Table 3. All the strains belonged to pv. *piscis*, most of them were positive for PHH-PCR *pilF* with the exception of the L5 isolate, and belonged to other serovars with the exception of the L1-clade A isolates, which belonged to Ser T.

**Table 3.** Genotypic and phenotypic characterization of selected strains from previously described lineages and/or clades possessing a pFv-related plasmid.

Lineage/clade^a^	Selected strain	Isolation data^b^		API20E profile^c^	PCR for^d^:					Serology^e^				
		Source	Year		species	pv. *piscis*	clade E	*ftbp*	PHH	SerE	SerA	SerI	SerO	SerT
L1-clade A	yb158	Healthy tilapia	2005	5146105	+	+	-	+	+	-	-	-	-	+
	V246	Human blood	2005	5146105	+	+	-	+	+	-	-	-	-	+
L3	12	Human blood	1996	4146005	+	+	-	+	+	-	-	-	+	-
L5	V252	Human blood	2004	5346005	+	+	-	+	-	-	-	-	-	-

^a^ Phylogenetic lineage determined by Roig et al [11]. L3 and L5 are clonal complexes.

^b^ Data of isolation for the selected strain

^c^ Probability of identification of *V. vulnificus*: 4146005, 75.5%; 5146105, 99.3%.

^d^ The target genes for PCR were: *vvhA*, species; *fpcrp*, pv. *piscis* (PCR designed in this work); *seq61*, zoonotic clade E [21]; *ftbp,* fish transferrin binding protein [12]; a *pilF* polymorphism, public health hazard (PHH) [22]. Discrimination is based on the amplification of a variable region located within the gene *pilF* resulting in a 338 bp fragment.

^e^ +, AgO agglutination in less than 1 min and ELISA titter (the reciprocal of the highest dilution of the antiserum giving OD two times higher than that of the negative control) higher than 60,000. -, no agglutination and ELISA titer lower than 15,000.

*Virulence assays: ex vivo and in vivo assays.* The strains resisted and multiplied in tilapia plasma, were virulent to tilapia by immersion and were able to grow in human serum plus iron (Table 4), confirming that they constituted new zoonotic groups within the pv. *piscis*.

**Table 4.** In vivo and ex vivo virulence assays performed with selected strains from previously described lineages and/or clades possessing a pFv-related plasmid**.**

Lineage/ clade^a^	Selected strain	Resistance to^b^		Virulence for^c^
		TP	HS + Fe	Tilapia
L1-clade A	yb158	+ (5.2 × 10^3^%)	+ (4.0 × 10^3^%)	+ (2.5 × 10^7^)
	V246	+ (1.5 × 10^3^%)	+ (1.6 × 10^3^%)	NT
L3	12	+ (7.1 × 10^3^%)	+ (7.3 × 10^2^%)	+ (1.3 × 10^7^)
L5	V252	+ (9.2 × 10^2^%)	+ (7.7 × 10^2^%)	+ (2.5 × 10^6^)

^a^ Phylogenetic lineage determined by Roig et al [11]. L3 and L5 are clonal complexes.

^b^ Resistance to tilapia plasma (TP) and iron-overloaded human serum (HS+ 10 μM of FeCl_3_ [13]) after 4 h at 28°C (tilapia) or 6 h at 37°C (human) is coded as follow: +, survival ≥100%; -, survival <100%. Data in parentheses correspond to the medium percent survival from three independent experiments.

^c^ Virulence was determined by immersion challenge. The medium value of 50% lethal dose (LD_50_) from two different experiments is presented in parentheses as cfu/ml. Results are coded as follows: - (≥10^8^) +(< 10^8^) [10, 24].

NT, non tested.

*Genomic analysis*. We tested the hypothesis that L3, L1-clade A and L5 strains might harbour a virulence plasmid by searching for it in their genomes. Figure 2(B) shows the plasmids found in the selected strains. All the strains harboured a plasmid very similar to pCladeT. Moreover, pCladeA and pL5 were virtually identical to pCladeT, whereas pL3 differed mainly from the rest by the presence of a complete cluster of *tra* genes. More importantly, all the plasmids contained the virulence genes *fpcrp* and *ftbp* involved in fish septicaemia, which suggests that they were fish virulence plasmids (Figure 2B and Supplementary file 2). The presence of these genes was tested by PCR in all the strains from our collection belonging to L3, L5 and L1-clade A, and all of them were positive (Table 3) (Supplementary Figure 5). This result confirmed that similar plasmids were present in all these groups.

pCladeT, pCladeA, pL3 and pL5 lacked the *rtxA1* cluster. Therefore, we searched for it in their genomes and found it in ChrII. As can be seen in Figure 3(B), the toxins were more similar to each other and to MARTX_L1-clade T_ than to MARTX_L2-clade E_.

### Host specificity within pv. *piscis*

Since we had detected host specificity within pv. *piscis*, we analyzed the phylogenetic relationships of *fpcrp* and *ftbp*. The corresponding gene trees reconstructed from the orthologous genes found in the genomes used in [11] are shown in Figure 1. As expected, both genes were present in all the genomes belonging to pv. *piscis* as well as in a new L1 strain (FORC017) isolated from the blood of a woman infected after consuming raw fish [54]. Remarkably, the tree showed the strains grouped by host and not by phylogenetic group. Finally, both genes were also present in a *V. harveyi* strain, suggesting horizontal gene transfer (HGT) between two different species that share the same habitat.

## Discussion

To date, *V. vulnificus* has only been considered as a human pathogen linked to raw seafood ingestion or severe wound infection after seawater exposure but not as a true zoonotic agent linked to fish farms. The reason was probably that all zoonotic strains were a minor group and as such, they were seen as an anomaly in the species. However, in this paper we provide multiple pieces of evidence linking zoonosis to HGT in fish farms and demonstrating that L3 and L5, along with two clades present in L1 (clade A and clade T), probably arose following outbreaks of vibriosis in fish, as occurred with L2-clade E, -clade A and -clade I [8,10,23]. Although few strains from these lineages and clades have been analysed in the present work, they are clonal groups and the results obtained can probably be generalized to the clade/lineage level.

**Figure 4.**
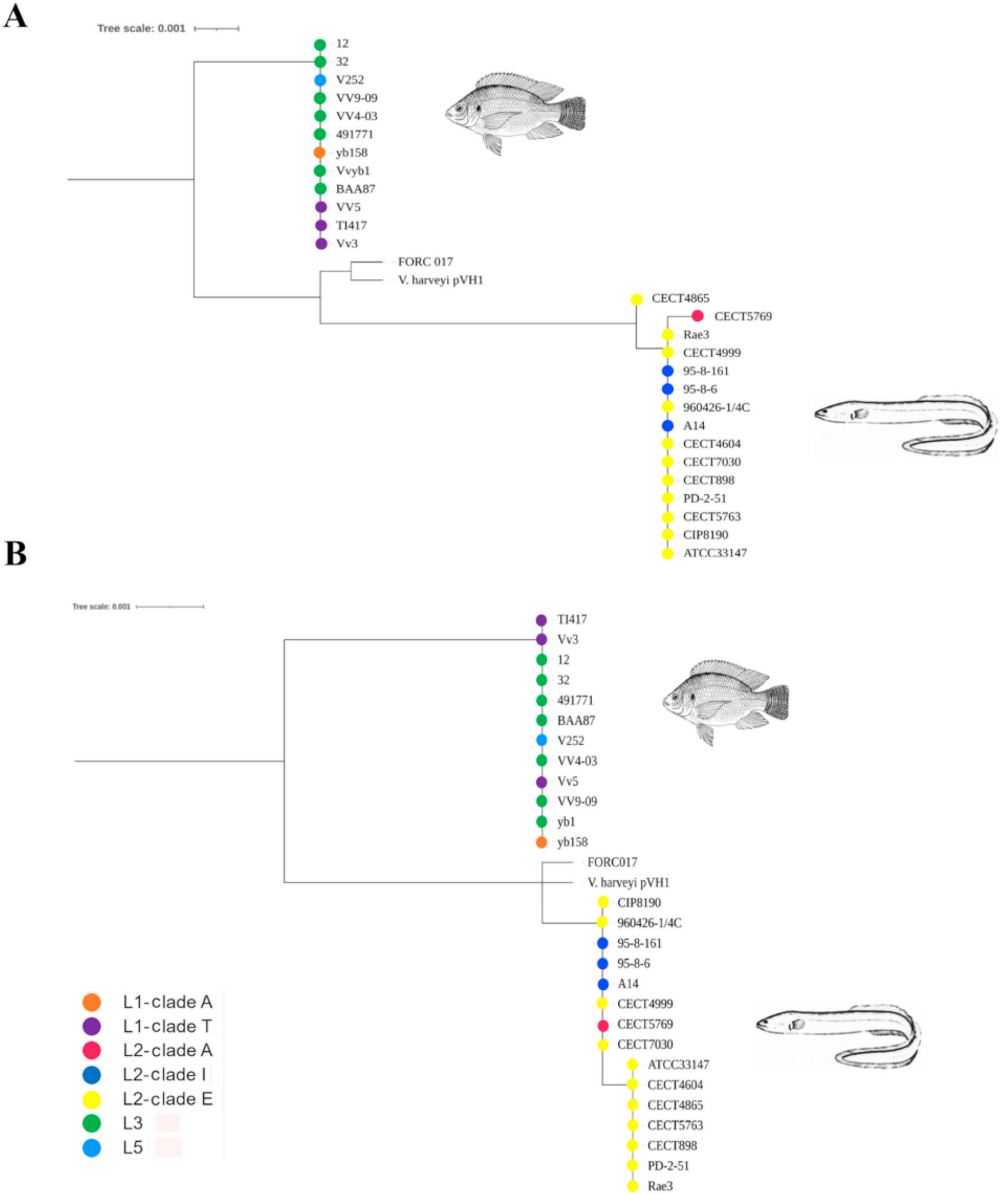
Evolutionary history of the genes for the “survival in fish blood kit”. Molecular phylogenetic analysis of *ftbp* **(A)** and *fpcrp* **(B)** was performed using the maximum likelihood method based on the Tamura 3-parameter model [47]. The tree is drawn to scale, with branch lengths measured as the number of substitutions per site. The main host (tilapia or eel) is shown in each tree.

The pieces of evidence are the following:

1. *The emergence of a new potentially zoonotic pv. piscis clade within L1*. A new clade emerged as a homogeneous and distinct serological group in tilapia farms located in the Eastern Mediterranean, where it caused several vibriosis outbreaks between 2016 and 2019. Genomic and phylogenomic analyses of L1-clade T strains revealed that they were highly homogeneous and formed a clonal group that was identified as belonging to pv. *piscis*. Unexpectedly, the new clade did not belong to L2, the lineage encompassing all the strains of pv. *piscis* known to date, but to L1, the lineage containing most of the strains associated with primary sepsis after shellfish ingestion. Similarly, to L2-clade E, L1-clade T resulted to be potentially zoonotic as it was virulent to mice, tested positive in the PCR designed to predict public health hazard [22], and, most importantly, multiplied in iron-overloaded human serum. This result was shared with all the human clinical strains used in this study as controls.
2. *The strains of the new clade share more virulence traits with phylogenetically distant strains but from the same habitat (tilapia farms) and location (Eastern Mediterranean) than with phylogenetically closer strains but from other sources and locations (e.g. YJ016 or CMCP6).* Among the shared virulence traits by Eastern Mediterranean lineages and clades, the following should be highlighted.
  - O-serogroup. Clade A and clade T shared O-serogroup despite belonging to two different sublineages within L1, which is compatible with an LPS-biosynthetic gene transfer between the two sublineages. Since the O-antigen confers partial resistance to fish serum in *V. vulnificus* [55], these events could have been favoured by positive selection of resistant strains in the fish farming environment.
  - MARTX toxin. These toxins are the most important virulence factors in *V. vulnificus* regardless of lineage and susceptible host [14,50,51]. At least 7 types of these toxins and 8 functional domains have been described in this species [50]. The pv. *piscis* strains studied to date produce a type known as RtxA1_3_ (MARTX_L2-clade E_ in this study), which has been implicated in toxic shock death in mice and eels [14,51]. A distinguishing feature of this toxin is the presence of an actin cross-linking domain (ACD). The *in-silico* analysis performed in this study revealed that the L1-clade T toxin is much more similar to the L3, L5 and L1-clade A toxins than to MARTX_L2-clade E_ (Figure 3). Like MARTX_L3_, it lacks the ACD domain and contains the RID, ABH, DUF1 and ExoY domains, although it lacks the DmX domain, a domain that disrupts the Golgi apparatus [56]. Laboratory experiments have shown that the variability of these toxins arises by recombination between two non-identical *rtxA1* genes carried by the same cell after mating between inter-domain homologous zones [57]. Therefore, this recombination process associated with HGT events is likely to have occurred multiple times in tilapia farms, with the most successful forms being selected.
  - The outer membrane proteins Fpcrp and Ftbp. Both proteins protect the bacterium against innate immunity in eel blood [12,50]. The genes *fpcrp* and *ftbp* were present in all Eastern Mediterranean lineages and clades, and its phylogenetic analysis revealed small differences that could be related to host adaptation as the strains grouped by host fish species (Figure 4). Approximately 13% of salmonid transferrin sequences have been shown to undergo positive selection for iron competition with bacterial pathogens [58]. Similarly, pathogens can change amino acids in their transferrin receptors through mutations that facilitate the adaptation of these bacteria to changes in the host or even to new hosts [58].
  - Zoonotic capability. All the Eastern Mediterranean lineages and clades were zoonotic or potentially zoonotic as they included human clinical strains [9,17,18] and, at the same time, were virulent to tilapia.
3. The four clades/lineages associated with Eastern Mediterranean tilapia farms presented a new pFv-related plasmid that could have emerged from recombination between pFv and pConj. The new plasmids were practically identical, and the main difference was that pL3 was the only one containing a complete set of tra genes for conjugal transference. Recombination between pFv and pConj had been previously demonstrated at the laboratory scale by Lee et al. [49] but it had not been shown to occur in nature. It therefore seems likely that pCladeA, pCladeT, pL3, pL5 and pFv belong to the same family of fish virulence plasmids, a family that could have spread to four of the five lineages described in the species.

Finally, the Eastern-Mediterranean isolates were virulent for tilapia but not for eel, which suggests a specific host adaptation that is supported by the differences in *ftbp* and *fpcrp* sequences (Figure 4). Remarkably, the genomic analysis in the context of the genus also revealed that plasmid-encoded genes *ftbp* and *fpcrp* have already been transmitted to another pathogenic fish species, *V. harveyi.* The relevance of this acquisition for fish virulence in this species is being studied now.

In summary, our work provides multiple pieces of evidence supporting the hypothesis that *V. vulnificus* is an underestimated zoonotic agent linked to fish farms. The species is probably an opportunistic pathogen in its natural environment, but in fish farms some strains behave as primary fish pathogens after acquiring a plasmid that encodes the ability to proliferate in the fish blood. Natural selection has probably favoured the amplification of some transformant clones after successive vibriosis outbreaks, giving rise to the clades that are isolated nowadays. One of these clades (L2-clade E) has been reported as a zoonotic agent [10], while others are probably linked to cases of unreported zoonoses or even zoonotic outbreaks (L3). It is of concern that none of these groups, especially L3, has been associated with outbreaks of vibriosis in tilapia. The consequence is that this species is probably being underestimated as a zoonotic pathogen. Further phenotypic, genomic and phylogenomic analyses of new isolates of this species will be necessary to confirm that fish farms are acting as drivers accelerating the evolution of *V. vulnificus*.

***Ethics statement.*** All assays involving rabbit/mice/fish were approved by the Institutional Animal Care and Use Committee and the local authority (Conselleria de Agricultura, Medio Ambiente, Cambio Climático y Desarrollo Rural. Generalitat Valenciana), following European Directive 2010/63/EU and the Spanish law “Real Decreto 53/2013.” Murine and fish infections were performed under the project licenses 2016/VSC/PEA/00069, 2017/VSC/PEA/00053and 2020/VSC/PEA/0202 in the Research Animals core and Fish (code ES461900001203) facilities of the University of Valencia (UV), respectively. Rabbit immunization was performed under the project license 2016/VSC/PEA/00073 and 2020/VSC/PEA/0054 in the facility of the UV.

## Supplementary Material

Supplementary_file_2.xlsx

Supplementary_file_1.docx

Supplementaryfig6.png

Supplementaryfig5.png

Supplementaryfig4.png

Supplementaryfig3.png

Supplementaryfig2.png

Supplementaryfig1.png

Supplementary_Table_2.docx

Supplementary_table_1.docx
