# Supplementary material for: The widespread presence of a family of fish virulence plasmids in Vibrio vulnificus stresses its relevance as a zoonotic pathogen linked to fish farms
Source: Emerg Microbes Infect. 2021 Nov 18;10(1):2128–40. doi: 10.1080/22221751.2021.1999177 (PMC8635547; doi:10.1080/22221751.2021.1999177)
Supplement: Supplementary_file_1.docx [file TEMI_A_1999177_SM5837.docx]

**Supplementary Material 1**

**Material and methods**

**Bacteria culturing and conservation**

Bacteria were routinely cultured in Tryptone Soy Agar (1%NaCl; TSA-1) at 28°C for 24 h. All strains were stored in Lauria Bertani broth (1% NaCl; LB-1) plus glycerol (17%) at -80°C.

***Disease description and sampling***

Three outbreaks affecting three Nile tilapia (*Oreochromis niloticus*) farms occurred in 2016 and 2019 in Eastern Mediterranean. Tilapias were reared in brackish water (0.2 % salinity) at temperatures of 26–27ºC. Mortality was low but continuous during the course of the outbreaks. Although the cumulative fish losses were lower than 5% of the stock in each epizootic, the disease proved costly because of the size of affected fish (150-400 g).

The most remarkable clinical signs were haemorragic areas in the head and at the base of rectal fin, exophtalmia and skin ulcers. Internally, the liver was pale and yellowish, the brain was oedematous and, in some cases, a fine layer of oily liquid covering the peritoneal cavity was observed. Each moribund fish was packed in a separate labelled plastic bag and transported to lab under refrigerated conditions. For bacterial isolation, samples aseptically collected from kidney, brain, eye and other damaged zones in the head were directly streaked onto salt blood agar base (Oxoid Ltd, Basingstoke, UK) with 5% citrated calf blood and 1% NaCl and plates were incubated at 28 º C for 48 h (1).

***Serological characterization***

*Antisera.* Rabbit antisera were prepared by intravenous injection of New Zealand rabbits with formalin-killed cells according to the procedure described by (2). One week after the last injection, the rabbits were bled from the ear vein. All sera were stored in aliquots and frozen at -80º C until used.

*Slide-agglutination test.* The slide agglutination assay was performed by mixing 10 μl of suspensions of O-antigens in PBS (pH 7.4) with 10 μl of rabbit polyclonal antisera (3). A distinct and rapid agglutination was defined as positive.

*Enzyme-linked immunosorbent assay (ELISA).* Suspensions of O-antigens in bicarbonate buffer (0.05 M, pH 9.6) were prepared and ELISA was performed as follows. 96-well microplate plates (Corne, USA) were coated with 50 μl of the O-antigen suspension and plates were incubated overnight at 4 °C. Unbound antigens were removed by washing 3 times with PBS-T (0.1% Tween in phosphate buffered saline, pH=7.2). After blocking with 1% bovine serum albumin in PBS, serial dilutions of antisera (from 1:5000 to 1:100000) in PBS were placed and plates were incubated for 1.5 h at 37 °C. Wells were then washed 4 times with PBST and incubated with diluted secondary antibody, a diluted goat anti-rabbit antibody conjugated with horse radish peroxidase (Auragene Bioscience, Co., Ltd., Changsha, China. 1:10,000), for 1 h at 37 °C. Wells were then washed again 5 times with PBST. Finally, 50 μl of tetrabenzidine (TMB, substrate solution) was added and developed for 10 min at 25 °C. To stop the reaction, 50 μl of H_2_SO_4_ (2 M) was added to each well and the OD at 450 nm was measured. An OD value ≥ 2X the OD for the negative control (PBS) was considered as positive. Serum titer was calculated as the reciprocal of the highest antibody dilution giving a positive result.

***Maintenance of animals and virulence assays***

Juvenile healthy Nile tilapia (mean weight 8-10 g) were obtained from a Spanish fish farm and, prior to challenges, they were kept separately at 26 ºC for 7 days in glass tanks containing dechlorinated water supplemented with 0.5 % NaCl (w/v) supplied with filtration and air systems. Virulence for mice was determined in 6- to 8-week old female (mice BALB/c, Charles River, France).

Virulence for fish was tested by intraperitoneal (i.p.) injection and immersion challenge according to (4). In the case of i.p. infection, 0.1 ml of ten-fold serially diluted bacterial suspensions in PBS containing between 10^2^ and 10^7^ colony forming units (cfu) were injected per animal (6 animals per bacterial dose, in duplicate). In the case of bath challenges, groups of 10 tilapias were exposed separately for 60 min to serial dilutions containing between10^5^ and 10^8^ cfu/ml (two fish groups by dose). After challenge, each group of fish was placed in a new aquarium and the number of culturable cells in the challenge suspension was determined by drop plating on TSA-1. Virulence for mice was tested by i.p. injection according to (10). Bacterial doses ranging from 10^2^ to 10^8^ cfu per mouse were injected (6 animals per bacterial dose, in duplicate). Mortalities were recorded daily for 10 days only if the inoculated bacterium was recovered as pure culture and from internal organs of moribund animals and confirmed by serology to be the inoculated strain. Control groups of animals (in duplicate) were challenged with sterile PBS.

***Resistance to fish plasma and human serum***

For plasma extraction, healthy tilapias were anaesthetised in a benzocaine solution (0.02mg ml^-1^) for 5-10 minutes and blood was collected by caudal vessel puncture with heparinised syringe according to (5). Fish plasma samples and human serum, purchased from Sigma-Aldrich, were stored at -80ºC until use.

The survival in fresh tilapia plasma and sera (with and without FeCl_3_ 10 μm) was assayed with stationary-phase bacteria in microtitre plates. In each well, a volume of 20 μl serum was mixed with 20 μl of a suspension of bacteria (10^4^–10^5^ cfu/ml) in sterile saline solution [0.9% (w/v) NaCl, pH 7, SS]. The assays were performed in triplicate and samples were taken at 0 h and at 4 h (fish) or 6 h (human) post-incubation at 28 ºC (fish plasma assay) or 37 ºC (human serum assay). Viable counts were determined by drop plating on TSA-1. Bactericidal and bacteriostatic activities in serum were measured as the percentage survival of the strains in these fluids after 4 or 6 h of incubation, depending of the assay.

1. Fouz B, Larsen JL, Amaro C. Vibrio vulnificus serovar A: an emerging pathogen in European anguilliculture. J Fish Dis. 2006 May;29(5):285–91.

2. Sørensen UB, Larsen JL. Serotyping of Vibrio anguillarum. Appl Environ Microbiol. 1986;51(3):593–7.

3. Fouz B, Alcaide E, Barrera R, Amaro C. Susceptibility of Nile tilapia (Oreochromis niloticus) to vibriosis due to Vibrio vulnificus biotype 2 (serovar E). Aquaculture. 2002 Sep 23;212(1–4):21–30.

4. Amaro C, Biosca EG, Fouz B, Alcaide E, Esteve C. Evidence that water transmits Vibrio vulnificus biotype 2 infections to eels. Appl Environ Microbiol. 1995;61(3):1133–7.

5. Amaro C, Biosca EG. Vibrio vulnificus biotype 2, pathogenic for eels, is also an opportunistic pathogen for humans. Appl Environ Microbiol. 1996 Apr;62(4):1454–7.
