# Supplementary figures and images for: The widespread presence of a family of fish virulence plasmids in Vibrio vulnificus stresses its relevance as a zoonotic pathogen linked to fish farms
Source: Emerg Microbes Infect. 2021 Nov 18;10(1):2128–40. doi: 10.1080/22221751.2021.1999177 (PMC8635547; doi:10.1080/22221751.2021.1999177)

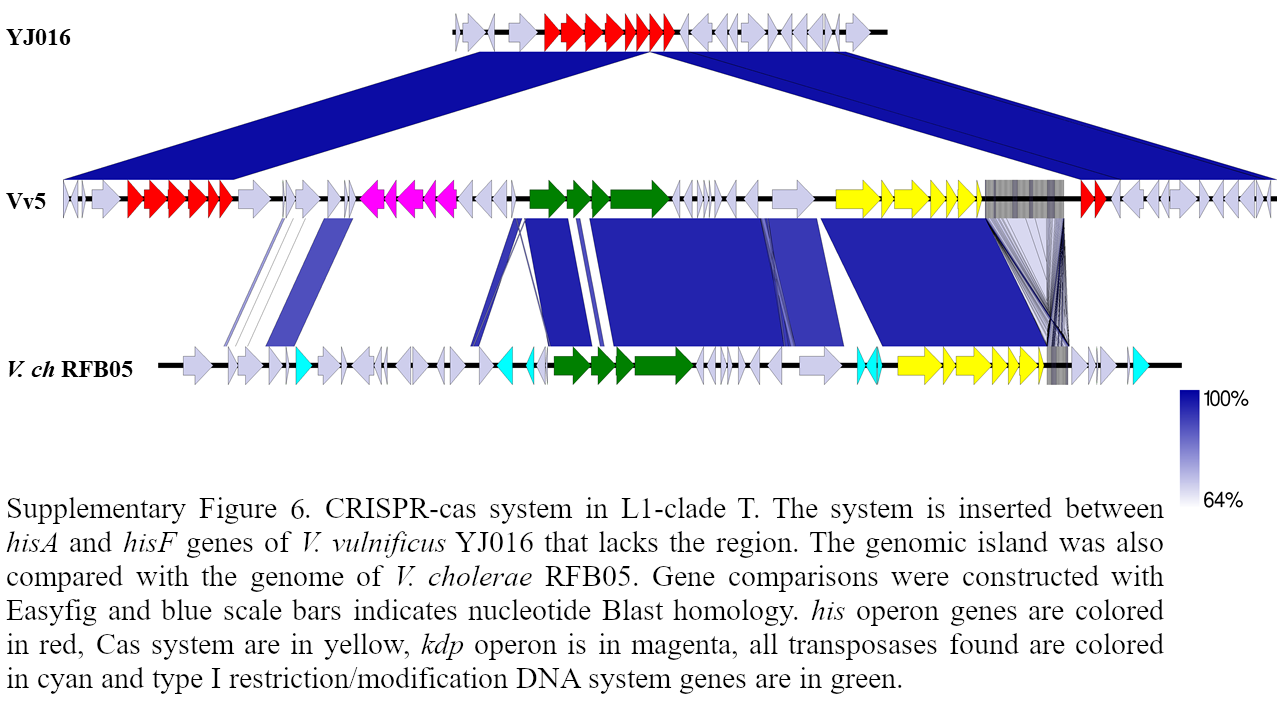

Supplement: Supplementaryfig6.png [file TEMI_A_1999177_SM5836.png]

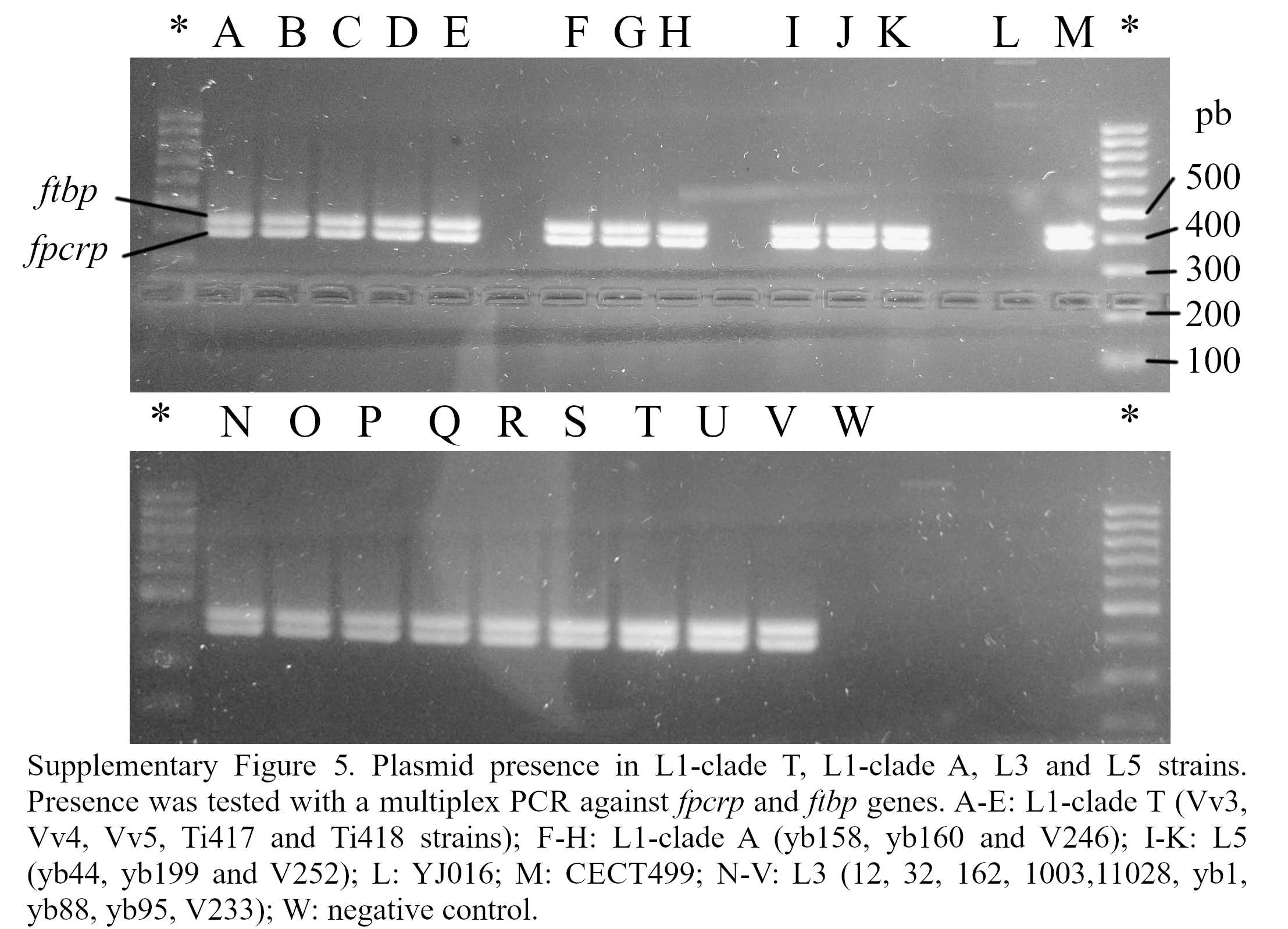

Supplement: Supplementaryfig5.png [file TEMI_A_1999177_SM5835.png]

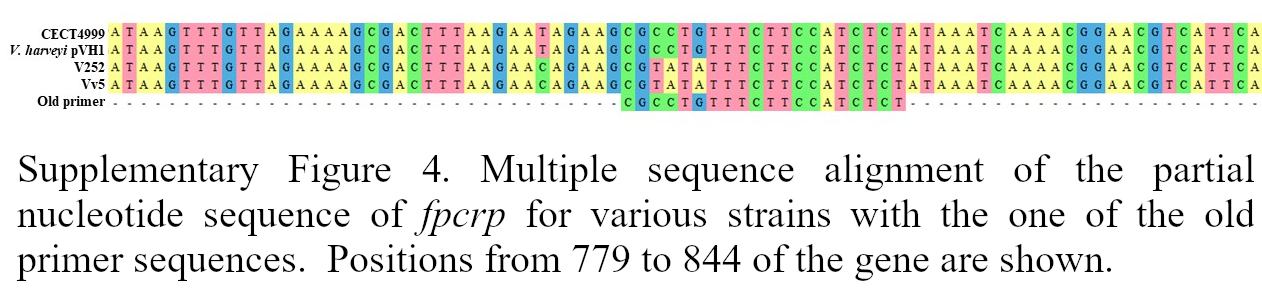

Supplement: Supplementaryfig4.png [file TEMI_A_1999177_SM5834.png]

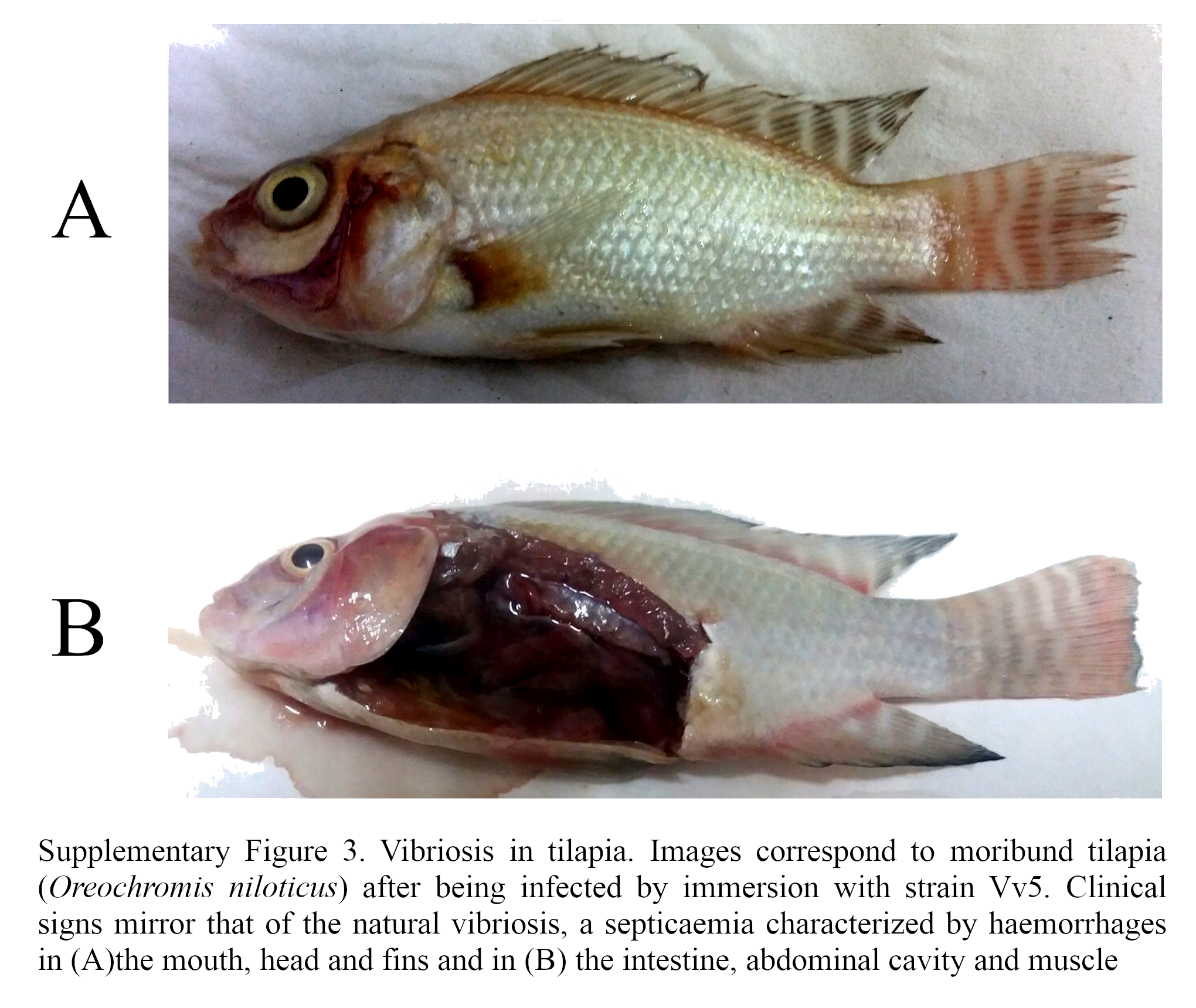

Supplement: Supplementaryfig3.png [file TEMI_A_1999177_SM5833.png]

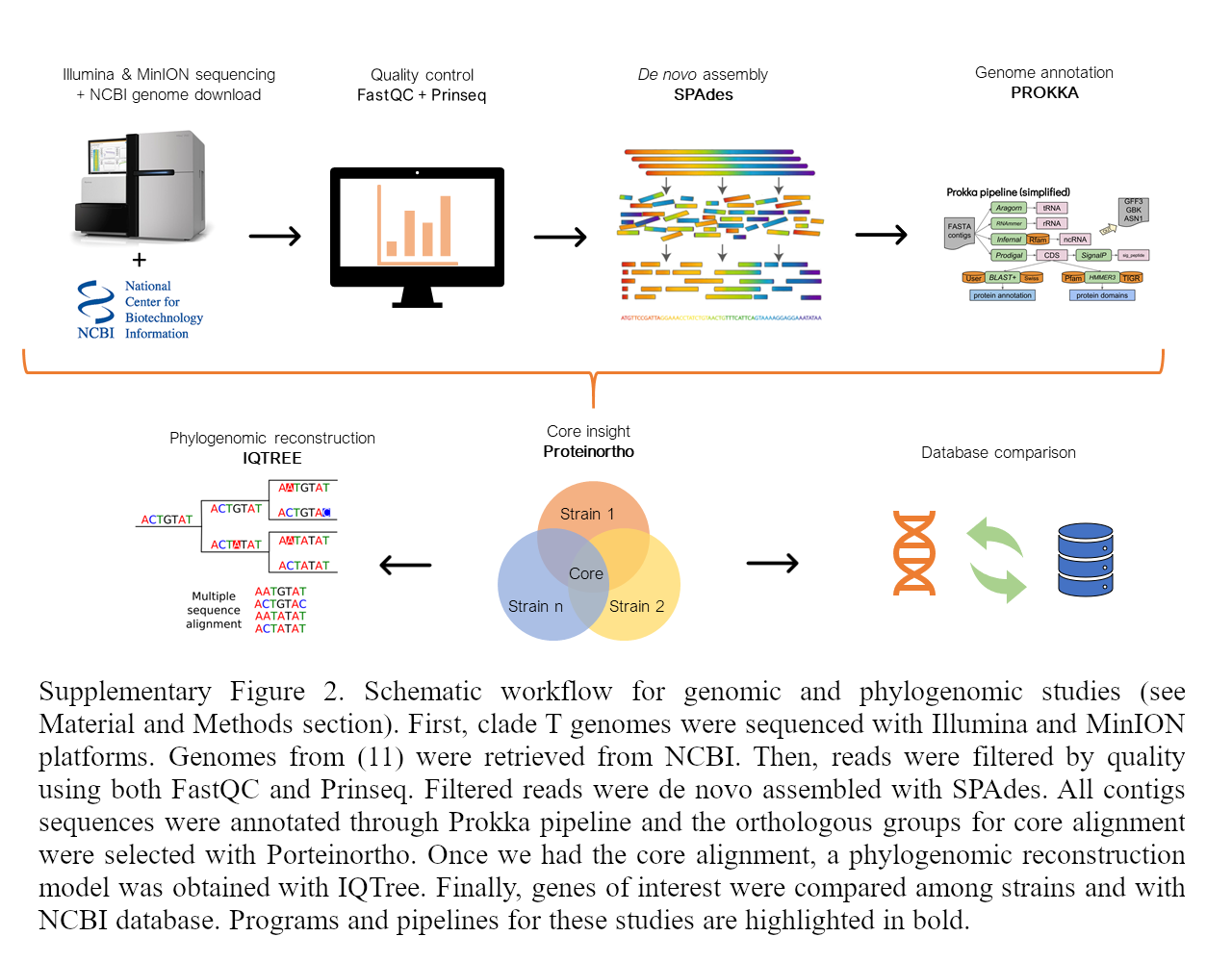

Supplement: Supplementaryfig2.png [file TEMI_A_1999177_SM5832.png]

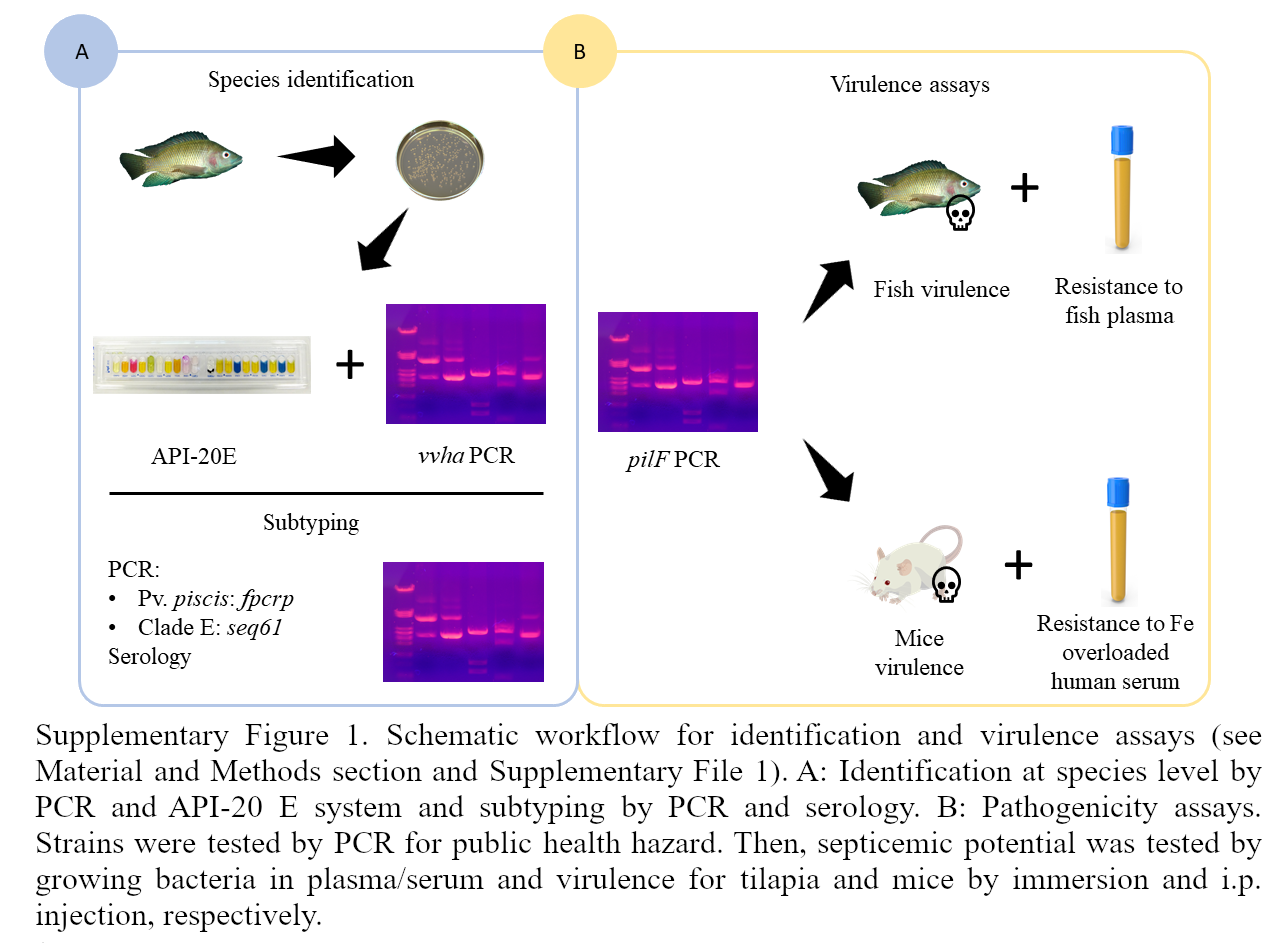

Supplement: Supplementaryfig1.png [file TEMI_A_1999177_SM5831.png]
