# Supplementary material for: The widespread presence of a family of fish virulence plasmids in Vibrio vulnificus stresses its relevance as a zoonotic pathogen linked to fish farms
Source: Emerg Microbes Infect. 2021 Nov 18;10(1):2128–40. doi: 10.1080/22221751.2021.1999177 (PMC8635547; doi:10.1080/22221751.2021.1999177)
Supplement: Supplementary_Table_2.docx [file TEMI_A_1999177_SM5830.docx]

**Supplementary Table 2.** ANI values using Clade T VV5 strain as a reference.

| Lineage/pathovar | Strain | ANI value |
| --- | --- | --- |
| 1 | Vv3 | 99.97 |
| 1 | TI417 | 99.95 |
| 1 | Yb158 | 98.20 |
| 1 | CMCP6 | 98.18 |
| 1 | YJ016 | 98.10 |
| 2/pv. *piscis* | CECT5769 | 95.43 |
| 2/pv. *piscis* | CECT4999 | 95.37 |
| 2/pv. *piscis* | 95-8-161 | 95.31 |
| 3 | BAA87 | 96.76 |
| 4 | Riu1 | 97.12 |
| 5 | V252 | 96.22 |
