# Supplementary material for: The widespread presence of a family of fish virulence plasmids in Vibrio vulnificus stresses its relevance as a zoonotic pathogen linked to fish farms
Source: Emerg Microbes Infect. 2021 Nov 18;10(1):2128–40. doi: 10.1080/22221751.2021.1999177 (PMC8635547; doi:10.1080/22221751.2021.1999177)
Supplement: Supplementary_table_1.docx [file TEMI_A_1999177_SM5829.docx]

**Supplementary Table 1. Serological relationships among the *V. vulnificus* strains.**

| **Strain (serovar)^a^** | Slide agglutination ^b^ | | | | | |  | | ELISA titer ^c^ | |
| --- | --- | --- | --- | --- | --- | --- | --- | --- | --- | --- |
|  | VV3 | VV5 | CECT4999 | CECT5198 | 95-8-161 | VV12 |  | VV3 | | VV5 |
| **New isolates** |  |  |  |  |  |  |  |  | |  |
| VV3 | ++ | ++ | - | - | - | - |  | +++ | | +++ |
| VV4 | ++ | ++ | - | - | - | - |  | +++ | | +++ |
| VV5 | ++ | ++ | - | - | - | - |  | +++ | | +++ |
| TI417 | ++ | ++ | - | - | - | - |  | +++ | | +++ |
| **Control strains** |  |  |  |  |  |  |  |  | |  |
| CECT 529^T^ (ND) | - | - | - | - | - | - |  | - | | - |
| YJ016 (ND) | - | - | - | - | - | - |  | - | | - |
| CECT 4999 (SerE) | - | - | ++ | - | - | - |  | - | | - |
| CECT 5198 (SerA) | - | - | - | ++ | - | - |  | - | | - |
| 95-8-161 (SerI) | - | - | - | - | ++ | - |  | - | | - |
| VV12 (SerO) | - | - | - | - | - | ++ |  | - | | - |
| yb158 (ND) | + | + | - | - | - | - |  | ++ | | ++ |
| V246 (ND) | + | + | - | - | - | - |  | ++ | | ++ |
| V252 (ND) | - | - | - | - | - | - |  | - | | - |

^a^ CECT, Spanish Type Culture Collection; T, type strain.

^b^Slide-agglutination were performed by mixing appropriate dilutions of polyclonal antibodies against whole cells (23) and O-antigens. ++, immediate agglutination; +, agglutination in less than 1 min.; -, no agglutination.

^c^ Serial two fold dilutions of O-antigens were mixed with appropriate dilutions of polyclonal antiserum in microtiter plates (23). ELISA titer was defined as the reciprocal of the highest dilution of the antiserum giving OD two times higher than that of the negative control and results were scored as follows: +++ >80,000; 80,000> ++ > 40,000; 40,000> + > 15,000; -, <15,000.

NT, non tested
